# Theobromine consumption does not improve fasting and postprandial vascular function in overweight and obese subjects

**DOI:** 10.1007/s00394-018-1612-6

**Published:** 2018-01-12

**Authors:** Lotte Smolders, Ronald P. Mensink, Jose J. van den Driessche, Peter J. Joris, Jogchum Plat

**Affiliations:** Department of Human Biology and Movement Sciences, School of Nutrition and Translational Research in Metabolism (NUTRIM), Maastricht University Medical Center, Universiteitssingel 50, P.O. Box 616, 6200 MD Maastricht, The Netherlands

**Keywords:** Theobromine, Endothelial function, Arterial stiffness, Microvasculature, Postprandial

## Abstract

**Backgound:**

Theobromine, a component of cocoa, may favorably affect conventional lipid-related cardiovascular risk markers, but effects on flow-mediated dilation (FMD) and other vascular function markers are not known.

**Objective:**

To evaluate the effects of 4-week theobromine consumption (500 mg/day) on fasting and postprandial vascular function markers.

**Design:**

In a randomized, double-blind crossover study, 44 apparently healthy overweight (*N* = 30) and obese (*N* = 14) men and women with low HDL-C concentrations, consumed daily 500 mg theobromine or placebo for 4 weeks. After 4 weeks, FMD, peripheral arterial tonometry (PAT), augmentation index (AIx), pulse wave velocity (PWV), blood pressure (BP) and retinal microvasculature measurements were performed. These measurements were carried out under fasting conditions and 2.5 h after a high-fat mixed meal challenge.

**Results:**

4-week theobromine consumption did not change fasting vascular function markers, except for a decrease in central AIx (cAIx, − 1.7 pp, *P* = 0.037) and a trend towards smaller venular calibers (− 2 µm, *P* = 0.074). Consuming a high-fat mixed meal decreased FMD (0.89 pp, *P* = 0.002), reactive hyperemia index (RHI, − 0.30, *P* < 0.001), peripheral systolic BP (SBP, − 3 mmHg, *P* ≤ 0.001), peripheral diastolic BP (DBP, − 2 mmHg, *P* ≤ 0.001), central SBP (− 6 mmHg, *P* ≤ 0.001) and central DBP (− 2 mmHg, *P* ≤ 0.001), but increased heart rate (HR, 2 bpm, *P* < 0.001). Theobromine did not modify these postprandial effects, but increased postprandially the brachial artery diameter (0.03 cm, *P* = 0.015), and decreased the cAIx corrected for a HR of 75 (cAIx75, − 5.0 pp, *P* = 0.004) and peripheral AIx (pAIx, − 6.3 pp, *P* = 0.017).

**Conclusion:**

Theobromine consumption did not improve fasting and postprandial endothelial function, but increased postprandial peripheral arterial diameters and decreased the AIx. These findings do not suggest that theobromine alone contributes to the proposed cardioprotective effects of cocoa.

This trial was registered on clinicaltrials.gov under study number NCT02209025.

**Electronic supplementary material:**

The online version of this article (10.1007/s00394-018-1612-6) contains supplementary material, which is available to authorized users.

## Introduction

Chocolate consumption is associated with a lower risk for cardiovascular diseases (CVD) [1]. These effects are specifically evident for dark chocolate, which has been shown to improve serum lipid profiles [2, 3], to reduce blood pressure [2, 4], to increase insulin sensitivity, and to improve vascular endothelial function as measured with flow-mediated dilation (FMD) [2]. The components from cocoa responsible for the potentially beneficial effects on FMD are unknown, but it can be argued that theobromine (500–700 mg/100 g dark chocolate) contributes to these effects. In fact, the acute consumption of low amounts of theobromine (111 mg) and caffeine (11 mg) potentiated the protective effect of cocoa flavanols on postprandial FMD [5]. Furthermore, a 4-week study showed that daily consumption of 500 or 850 mg theobromine lowered fasting low-density lipoprotein cholesterol (LDL-C) and apolipoprotein B (apoB) concentrations, and increased those of serum HDL-C [6, 7] and apoA-I [6]. In contrast to these studies, we recently reported that we could not fully confirm the findings on apoA-I concentrations [7]. Moreover, we observed no effects of a daily theobromine consumption of 500 mg on postprandial lipid responses, while postprandial free fatty acid, glucose and insulin responses were increased [7]. It is known that both postprandial hyperlipidemia [8] and hyperglycemia [9] impair vascular function. Therefore, it is relevant to examine effects of theobromine, not only on fasting vascular function, but also on vascular resilience after a meal challenge.

FMD is a non-invasive vascular function marker to assess endothelial function and an accepted predictive biomarker for future CVD events [10]. Another method to evaluate endothelial function is peripheral arterial tonometry (PAT), which measures the reactive hyperaemia index (RHI) of the small arteries and is negatively correlated with the presence of CVD risk factors [11]. Furthermore, several non-invasive markers exist to assess arterial stiffness such as carotid-femoral pulse wave velocity (PWVcf) and the augmentation index (AIx). PWVcf is associated with a higher frequency of stroke, CVD and total mortality [12], while the AIx is associated with higher CVD risk [13]. Finally, the microvasculature can be studied by evaluating the caliber of the blood vessels in the retina. Cross-sectional wider venules and narrower arterioles predict an increased risk of CVD events in women, but not in men [14].

In agreement with effects on FMD [2], dark chocolate consumption also improved RHI [15]. However, effects on measures of arterial stiffness are conflicting. In one study, no effects were found on PWVcf [13], while in another study beneficial effects were found on Aix [16, 17]. Furthermore, Terai et al. showed no differences in arteriolar and venular width after short-term dark chocolate consumption [12].

So far, long-term effects of theobromine consumption on a wide panel of vascular function markers have never been studied. Therefore, we examined the effects of 4-week theobromine consumption (500 mg/day) on FMD, RHI, pulse wave velocity (PWV), AIx, and retinal microvasculature in fasting conditions and after a high-fat meal challenge.

## Materials and methods

### Study population

Details of this study have been published before [7]. Briefly, 44 healthy overweight (BMI 25–30 kg/m^2^; *n* = 30) or slightly obese (BMI 30–35 kg/m^2^; *n* = 14) men (45–70 years; *n* = 28) and women (50–70 years; *n* = 16) participated. During two screening visits, with an interval of ≥ 1-week, blood pressure was measured in fourfold using an Omron M7 (Omron Healthycare Europe B.V., Hoofddorp, the Netherlands). The first measurement was discarded and the last three measurements were averaged. Furthermore, a fasting blood sample was taken for analysis of serum total cholesterol, HDL-C, and plasma glucose concentrations. Inclusion criteria were: fasting serum HDL-C concentrations below the 50th percentile of the Dutch population (< 1.2 mmol/L for men and < 1.5 mmol/L for women) [18], fasting serum total cholesterol concentrations < 8.0 mmol/L, fasting plasma glucose concentrations < 7.0 mmol/L, and no use of lipid-lowering, anti-diabetic or anti-hypertensive medication or a medically prescribed diet. All participants gave their written informed consent before entering the study. This study was conducted according to the guidelines laid down in the Declaration of Helsinki. The study protocol was approved by the Medical Ethical Committee of the University Hospital Maastricht. The trail was registered at clinicaltrials.gov under study number NCT02209025.

### Study design and product

This study with a randomized, double-blind, cross-over design consisted of two intervention periods of 4-week separated by a 4-week washout period. Starting 2-week before the first intervention period and during the entire study, participants were instructed by a research dietician to avoid cocoa-containing products, for which they received a detailed list with products. Since theobromine is a metabolite of caffeine, the consumption of caffeine-containing drinks was restricted to a maximum of four cups a day and volunteers were instructed not to change their intake throughout the study. Subjects consumed in random order a drink (20 mL) enriched with theobromine (500 mg/day) or placebo every day during breakfast (Supplemental Table 1). Theobromine was obtained from Fagron (Uitgeest, the Netherlands) and drinks were produced and provided by Pharmavize (Mariakerke, Belgium).

### Test day and test meal

At the end of the 4-week intervention and placebo periods, subjects visited the University in fasting condition (no food or drinks, except water, 12-h before the visit). To minimize effects of the previous meal, we provided all subjects with the same commercially available lasagne (638 kcal, 28.4 g protein, 44.0 g carbohydrates and 37.6 g fat) the evening before each of the two test days. Furthermore, subjects were asked to avoid alcohol consumptions and strenuous activities 48-h before a visit.

In the morning, volunteers arrived at the Department by public transport or car, to standardize measurements as much as possible. Upon arrival and after a 10 min rest in supine position, vascular function measurements were performed in fasting conditions. Next, subjects were asked to consume a high-fat mixed meal (965 kcal, 17.9 g protein, 86.7 g carbohydrates, 60.6 g fat and 341 mg cholesterol), which was actually a shake prepared with regular food items bought in the local supermarket, together with their experimental drink, within 10 min. For the next 2.5-h following the meal, participants were not allowed to eat or drink anything except water. After 2.5-h (T150), the same panel of vascular function measurements was performed in the same order, using the same protocols.

### Vascular measurements

Investigators were blinded during the study and data analyses. All vascular measurements were performed in a quiet and temperature-controlled (22 °C) room. Peripheral systolic blood pressure (pSBP), peripheral diastolic blood pressure (pDBP), FMD, RHI, PWVcf, AIx and retinal vascular image measurements were determined as described before. Furthermore, carotid-radial PWV (PWVcr) was measured with the SphygmoCor, as described for the PWVcf [19]. Central systolic blood pressure (cSBP) and central diastolic blood pressure (cDBP) values were obtained from the SphygmoCor measurements.

### Statistical analysis

Before the start of the study, it was calculated that the statistical power to detect a true difference of at least 1.20% points (pp) in FMD between the experimental and control period was over 80% (*α* = 0.05), when 43 subjects successfully completed the study. For these calculations, a within-subject variability of 2.82 pp in FMD [20] was used. As the expected drop-out rate was 10%, the aim was to recruit 48 men and women.

All data is presented as mean ± SD unless indicated otherwise. All parameters were checked for normal distributions with the Shapiro–Wilk test. Fasting measurements after 4-week of placebo or theobromine intervention were compared with the general mixed model procedure with subject as random factor, and treatment and period as fixed factors. Differences in postprandial changes after 4 weeks of placebo or theobromine interventions were also evaluated with general mixed models with subject as random factor and treatment and meal as fixed factors and a treatment × meal interaction. If this treatment × meal interaction was not significant, it was omitted from the model. Results were considered statistically significant if *P* ≤ 0.05. All statistical analyses were performed using SPSS 20.0 for Mac (SPSS Inc., Chicago, IL, USA).

## Results

### Study participants

After screening, 48 subjects were eligible for participation and started the study. During the first intervention period, four participants (one male and three female) discontinued the study. Thus, 44 participants completed the study. The flow diagram and subject characteristic are presented in supplemental Fig. 1 and supplemental Table 2. RHI data was missing for three persons, due to technical problems. For two subjects (one man, one woman), T0 values were missing and for one male participant, a T150 value was absent. For four persons CRAE and CRVE calibers were missing (one man and three women) and for two persons (one man and one woman) the T150 values were missing, because of a poor quality of the fundus photos.

### Fasting vascular function

In the fasting condition, theobromine consumption did not change FMD, brachial artery diameter, and RHI. Furthermore, PWVcf, PWVcr, pAIx and cAIx75 did not change, but the cAIx was significantly lower after theobromine intake (− 1.7 pp, 95% CI − 6.1, − 0.2, *P* = 0.037). The CRAE and AVR also remained stable during the study, but theobromine intake tended to decrease the CRVE (− 2 µm, 95% CI − 4, 0, *P* = 0.074). Finally, fasting BP and HR were not affected (Table 1).

**Table 1:** Brachial diameter, brachial artery FMD, RHI, PWVcf, PWVcr, cAIx, cAIx75, pAIx, CRAE, CRVE, AVR, pSBP, pDBP, cSBP, cDBP and HR in fasting (T0) and postprandial (T150) condition after 4 weeks of placebo or theobromine consumption

	Placebo			Theobromine		
	T0	T150	Change	T0	T150	Change
Brachial diameter (cm)	0.49 ± 0.06	0.50 ± 0.08	0.00 ± 0.04	0.49 ± 0.07	0.52 ± 0.08	0.03 ± 0.04*
Brachial artery FMD (%)^$^	4.87 ± 2.54	3.87 ± 2.32	− 1.00 ± 2.97	4.43 ± 2.01	3.65 ± 2.25	− 0.78 ± 2.48
RHI^1,$^	2.64 ± 0.68	2.38 ± 0.61	− 0.24 ± 0.65	2.58 ± 0.61	2.23 ± 0.47	− 0.35 ± 0.60
PWVcr (m/s)	7.1 ± 1.1	7.1 ± 1.1	− 0.1 ± 1.2	7.4 ± 1.3	7.1 ± 1.5	− 0.3 ± 1.6
PWVcf (m/s)	9.0 ± 1.4	9.0 ± 1.6	0.0 ± 1.3	8.8 ± 1.6	9.0 ± 1.5	0.2 ± 1.5
cAIx (%)	28.3 ± 9.9	21.9 ± 10.5	− 6.4 ± 6.2	26.6 ± 10.4^#^	15.2 ± 11.2	− 11.3 ± 8.4
pAIx (%)	− 14.8 ± 14.9	− 24.1 ± 13.7	− 9.3 ± 10.6	− 16.8 ± 15.3	− 32.4 ± 13.8	− 15.6 ± 14.4*
cAIx75 (%)	21.6 ± 8.7	16.3 ± 9.6	− 5.3 ± 6.5	21.4 ± 9.3	11.2 ± 10.7	− 10.3 ± 8.2*
CRAE (µm)^3^	135 ± 19	135 ± 19	0 ± 9	134 ± 19	136 ± 19	2 ± 6
CRVE (µm)^3^	230 ± 14	231 ± 13	0 ± 5	228 ± 14	231 ± 13	2 ± 7
AVR^3^	0.59 ± 0.09	0.59 ± 0.09	0.00 ± 0.05	0.58 ± 0.09	0.59 ± 0.09	0.01 ± 0.04
pSBP (mmHg)^$^	134 ± 14	132 ± 12	− 3 ± 9	134 ± 14	130 ± 13	− 4 ± 10
pDBP (mmHg)^$^	85 ± 10	83 ± 8	− 2 ± 5	86 ± 10	83 ± 9	− 3 ± 6
cSBP (mmHg)^$^	126 ± 13	121 ± 11	− 5 ± 8	125 ± 12	118 ± 13	− 7 ± 10
cDBP (mmHg)^$^	86 ± 9	84 ± 9	− 2 ± 6	87 ± 9	84 ± 9	− 3 ± 5
HR (bpm)^$^	62 ± 9	64 ± 10	2 ± 4	62 ± 8	65 ± 10	3 ± 7

Values are mean ± SD. *n* = 44. Linear mixed models were conducted to find significant differences

*FMD* flow mediated dilation, *RHI* reactive hyperemia index, *PWV* pulse wave velocity, *PWVcf* PWV of the carotis-femoralis, *PWVcr* PWV of the carotis-radialis, *cAIx* central augmentation index, *cAIx75* cAIx corrected for a heart rate of 75, *pAIx* peripheral augmentation index, *CRAE* mean arteriolar width, *CRVE* mean venular width, *AVR* arteriolar to venular ratio, *p* peripheral, *c* central, *SBP* systolic blood pressure, *DBP* diastolic blood pressure, *HR* heart rate

^#^*P* < 0.05 for fasting differences from placebo

* *P* < 0.05 for treatment × meal effects

^$^*P* < 0.05 for meal effects

^1^*n* = 42 at T0, *n* = 41 at T150 due to missing values

^2^*n* = 41 at T0, *n* = 39 at T150 due to missing values

### Postprandial vascular function

As expected, high-fat mixed meal intake significantly decreased FMD (− 0.89 pp, 95% CI − 1.43, − 0.35, *P* = 0.002) and RHI (− 0.30, 95% CI − 0.43, − 0.16, *P* < 0.001) responses, but these effects did not depend on theobromine consumption. However, the brachial artery diameter increased when theobromine was part of the test meal (0.03 cm, 95% CI 1.23, 4.45, *P* = 0.015 for treatment × meal effects).

Test meal consumption did not affect arterial stiffness as measured via PWVcf and PWVcr. These effects were not changed by theobromine consumption. However, theobromine consumption tended to decrease the postprandial cAIx (− 4.9 pp, 95% CI − 5.8, − 0.7, *P* = 0.080 for treatment × meal effects) and decreased the pAIx (− 6.3 pp, 95% CI − 9.2, − 2.4, *P* = 0.017 for treatment × meal effects) and cAIx75 (− 5.0 pp, 95% CI − 6.8, − 2.4, *P* = 0.004 for treatment × meal effects). Test meal consumption did not change retinal vascular calibers. Effects were not changed by theobromine consumption. Finally, the test meal significantly decreased cSBP (− 5 mmHg, 95% CI − 8, − 4, *P* ≤ 0.001), cDBP (− 2 mmHg, 95% CI − 3, − 1, *P* ≤ 0.001), pSBP (− 3 mmHg, 95% CI − 5, − 1, *P* ≤ 0.001) and pDBP (− 2 mmHg, 95% CI − 3, − 1, *P* ≤ 0.001) and increased HR (2 bpm, 95% CI 1, 3, *P* ≤ 0.001). These effects were not modified by theobromine (Table 1).

## Discussion

This randomized, double-blind, placebo-controlled intervention study showed that a daily intake of 500 mg theobromine for 4 weeks did not affect FMD, RHI, PWV and the retinal microvasculature in fasting and postprandial conditions. However, theobromine consumption increased brachial arterial diameters and decreased the AIx during the postprandial phase.

The amount of 500 mg theobromine provided corresponds to approximately 67–100 g of dark chocolate [21]. It has been shown that consumption of 100 g dark chocolate for 15 days increased fasting FMD by 1.5 pp [22], which has been explained by an increase in nitric oxide (NO) concentrations due to a higher endothelial-derived NO synthase activity [23]. Our study was adequately powered to detect such a change. Moreover, the finding that fasting RHI—which is also NO-mediated but more related to the small arteries and the microvasculature—did not change after theobromine consumption was also opposite to the effects observed after consuming cocoa [15]. Furthermore, theobromine did not modify the effects of a meal challenge on vascular resilience. It is well known that a meal high in fat or high in carbohydrates impairs endothelial function [24, 25]. During postprandial hyperlipemia and hyperglycemia, the production of reactive oxygen species increases, which decreases NO bioavailability and thereby endothelial function [24, 25]. Indeed, also our test meal stressed the endothelium, as evidenced by decreases in postprandial FMD and RHI values, which is in agreement with other studies [26–28]. In contrast to our results, flavanol-rich cocoa consumption ameliorated the decrease in FMD after intake of a fatty meal [29], while flavonoid-rich dark chocolate consumption even increased FMD values 1 h after intake [30]. Our data, therefore, suggests that the improvement in endothelial function after cocoa consumption is not solely due to the theobromine content of cocoa. Cocoa also contains other bioactive compounds that may affect FMD, such as epicatechin [2]. Furthermore, it is possible that synergistic effects of the different bioactive components in cocoa have caused the beneficial effects on FMD and RHI. Indeed, Sansone et al. have recently shown that a combination of theobromine (111 mg) and caffeine (11 mg) did not change the FMD, while flavanol consumption (820 mg) alone increased the FMD. When the flavanols were consumed together with the mixture of theobromine and caffeine, circulating concentrations of flavanol metabolites were increased, while the FMD improved even more [5].

Although theobromine did not change the FMD, we observed an unexpected increase in brachial artery diameters during the postprandial phase. Unfortunately, most studies investigating the effects of cocoa did not report effects on brachial artery diameters. However, one acute study showed an increase in the brachial artery diameter after flavonoid-rich dark chocolate consumption, but with a simultaneous increase in FMD values [30]. We can only speculate about the mechanism underlying the increase in brachial artery diameter. First, theobromine inhibits cyclic adenosinemonophosphate (cAMP)-phosphodiesterase [31, 32], which increases cellular cAMP levels. As a response, intracellular calcium concentrations may decrease, followed by dilatation of the skeletal muscle vasculature [33]. A second potential explanation relates to the increased postprandial insulin responses after theobromine consumption, as we have already earlier reported [7]. Insulin is known to cause vasodilatation of the larger arteries [34], leading to enlarged artery diameters.However, it is also possible that the totality of metabolic changes including elevated insulin, glucose and hsCRP in the postprandial phase after theobromine consumption as reported earlier [7], is the reason underlying the observed effects on the vasculature during the postprandial phase.

Theobromine did not change fasting and postprandial PWV, but decreased fasting and postprandial AIx. This suggests that effects on parameters reflecting arterial stiffness are divergent, as has also been reported in other studies [27, 35]. Differences in PWV are frequently caused by changes in blood pressure [36]. In agreement with the lack of effect on PWV, theobromine did not change fasting and postprandial blood pressure parameters. Neufingerl et al. also observed no effects of 4-week of theobromine consumption on fasting blood pressure [6]. Furthermore, cocoa consumption did not affect postprandial blood pressure [30, 37] and PWVcf values [30]. In contrast, the consumption of theobromine-enriched flavonoid-rich cocoa drink for 3 weeks increased fasting blood pressure and postprandial PWVcf, while it decreased postprandial blood pressure in hypertensive patients [38]. Possibly, the difference in theobromine dose and drink composition can explain the discrepancy with our findings, since van den Bogaard et al. used a daily theobromine intake of 979 mg, which was consumed in combination with flavonoids provided by the cocoa [38]. Structural characteristics of the vascular wall also determine PWV [39]. However, as both blood pressure and PWV did not change, it can be deduced that these characteristics were also not changed.

Unrelated to theobromine intake, we observed a postprandial decrease in blood pressure, but the anticipated decrease in postprandial PWV was not observed. However, effects of meal consumption on blood pressure as related to PWV are conflicting. One study has reported an increase in blood pressure and PWVcf after meal consumption [40]; another study a decrease in blood pressure, but no change in PWVcf [41], while no change in blood pressure but an increase in PWVcr has also been reported [42]. For now, it is not clear what causes the discrepancy between the different studies, but it may relate to differences in the amounts of fat in the test meals between the studies [40–42].

In our study, theobromine decreased fasting cAIx, but did not change fasting pAIx and cAIx75. This is in contrast with the effects of cocoa, since acute and 4-week dark chocolate consumption decreased fasting cAIx75 [16, 17]. Except for the effects on fasting AIx, the test meal with theobromine decreased cAIx75 and pAIx. This decrease may be related to the postprandial increase in peripheral artery diameters, as a blood vessel with a larger diameter causes a lower reflection wave, leading to a lower AIx. It can, therefore, be argued that our findings suggest that the main effect of theobromine is dilation of the small and medium-sized peripheral arteries in the postprandial state. If true, then it is unclear why the PWVcr—a measure for peripheral vascular stiffness—did not decrease after theobromine with meal consumption. Finally, theobromine and meal consumption did not affect the arteriolar and venular diameters in the fundus vasculature. Also, acute dark chocolate consumption had no effect on postprandial arteriolar and venular calibers [12].

In conclusion, theobromine consumption did not improve fasting and postprandial endothelial function, but increased postprandial peripheral arterial diameters and decreased the AIx. These findings do not suggest that theobromine alone contributes to the proposed cardioprotective effects of cocoa.

## Electronic supplementary material

Below is the link to the electronic supplementary material.

Supplementary material 1 (DOCX 76 KB)

## Abbreviations

AIx: Augmentation index
AVR: Arteriolar to venular ratio
BP: Blood pressure
cAIx: Central augmentation index
cAIx75: cAIx adjusted for a heart rate of 75
cDBP: Central diastolic blood pressure
CRAE: Central retinal arteriolar equivalent
CRVE: Central venular arteriolar equivalent
cSBP: Central systolic blood pressure
CVD: Cardiovascular disease
FMD: Flow-mediated dilation
HDL-C: High-density lipoprotein cholesterol
HR: Heart rate
LDL-C: Low density lipoprotein cholesterol
NO: Nitric oxide
pAIx: Peripheral augmentation index
PAT: Peripheral arterial tonometry
pDBP: Peripheral diastolic blood pressure
pp: Percentage point
pSBP: Peripheral systolic blood pressure
PWV: Pulse wave velocity
PWVcf: Carotid-femoral PWV
PWVcr: Carotid-radial PWV
RHI: Reactive hyperemia index
