# Supplementary material for: Theobromine consumption does not improve fasting and postprandial vascular function in overweight and obese subjects
Source: Eur J Nutr. 2018 Jan 12;58(3):981–7. doi: 10.1007/s00394-018-1612-6 (PMC6499748; doi:10.1007/s00394-018-1612-6)
Supplement: Supplementary file 1 — Supplementary material 1 (DOCX 76 KB) [file 394_2018_1612_MOESM1_ESM.docx]

# Supplementary data

Supplemental Table 1 Composition of the test drinks (20ml)

|  | Theobromine drink | Placebo drink |
| --- | --- | --- |
| Theobromine (mg) | 500 | - |
| Microcrystalline cellulose (mg) | - | 500 |
| Methyl cellulose (mg) | 150 | 150 |
| Sucralose (mg) | 10 | 10 |
| Sodium benzoate (mg) | 100 | 100 |
| Anise 0.1% (mg) | 20 | 20 |
| Water | Till 20 ml | Till 20 ml |


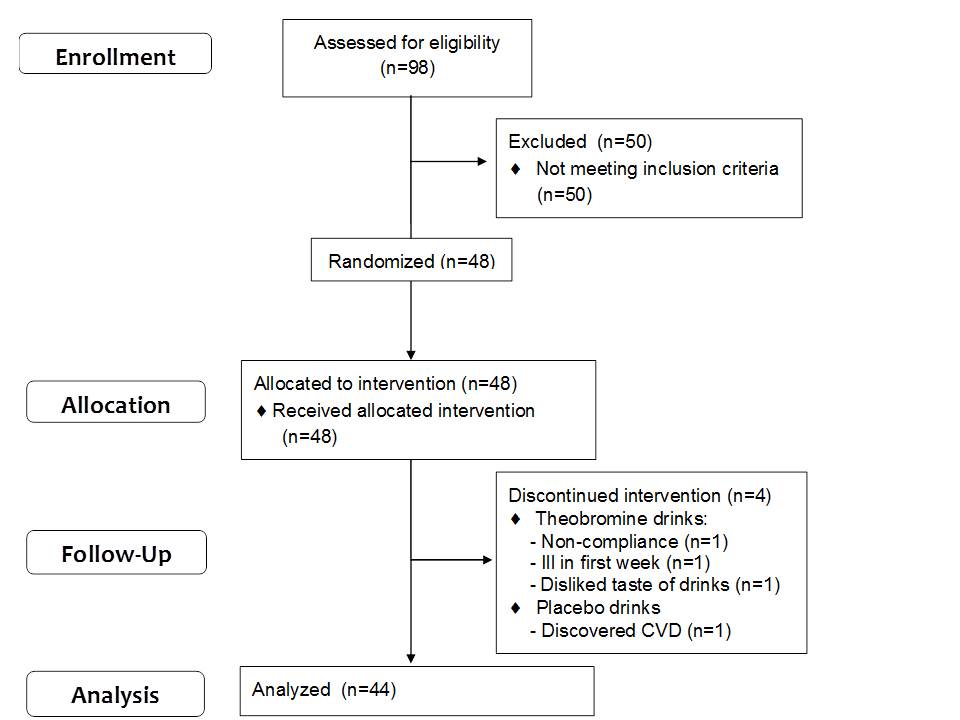


Supplemental Figure 1 Flow of participants throughout the study

Supplemental Table 2 Baseline characteristics of the participants that finished the study^1^

|  | Mean ± SD |
| --- | --- |
| Age (years) | 60.3 ± 5.5 |
| BMI (kg/m^2^)^1^ | 29.2 ± 3.0 |
| Total cholesterol (mmol/L) | 5.65 ± 0.92 |
| HDL-C (mmol/L)^1^ | 1.22 ± 0.18 |
| Glucose (mmol/L) | 5.56 ± 0.63 |
| SBP (mmHg)^1^ | 134 ± 15 |
| DBP (mmHg)^1^ | 86 ± 9 |
| Heart rate (bpm) | 70 ± 12 |

^1^ Values are mean ± SD. *n* = 44. BMI: Body mass index, SBP: systolic blood pressure, DBP: diastolic blood pressure.
